# Binary Toxin and Death after *Clostridium difficile* Infection

**DOI:** 10.3201/eid1706.101483

**Published:** 2011-06

**Authors:** Sabrina Bacci, Kåre Mølbak, Marianne K. Kjeldsen, Katharina E.P. Olsen

**Affiliations:** Author affiliations: European Programme for Intervention Epidemiology Training, Stockholm, Sweden (S. Bacci);; Statens Serum Institut, Copenhagen, Denmark (S. Bacci, K. Mølbak, M.K. Kjeldsen, K.E.P. Olsen)

**Keywords:** Clostridium difficile, bacteria, mortality, binary toxin, Denmark, Europe, case-fatality rate, surveillance, research

## Abstract

We compared 30-day case-fatality rates for patients infected with *Clostridium difficile* possessing genes for toxins A and B without binary toxin (n = 212) with rates for patients infected with *C. difficile* possessing genes for A, B, and binary toxin. The latter group comprised patients infected with strains of PCR ribotype 027 (CD027, n = 193) or non-027 (CD non-027, n = 72). Patients with binary toxin had higher case-fatality rates than patients without binary toxin, in univariate analysis (relative risk [RR] 1.8, 95% confidence interval [CI] 1.2–2.7) and multivariate analysis after adjustment for age, sex, and geographic region (RR 1.6, 95% CI 1.0–2.4). Similar case-fatality rates (27.8%, 28.0%) were observed for patients infected with CD027 or CD non-027. Binary toxin either is a marker for more virulent *C. difficile* strains or contributes directly to strain virulence. Efforts to control *C. difficile* infection should target all virulent strains irrespective of PCR ribotype.

## Abstract 2

TOC Summary: Strains with these genes in addition to toxins A and B were associated with the highest case-fatality rates.

*Clostridium difficile* infection (CDI) is a common cause of health care–associated diarrhea in industrialized countries (*1*), and the leading cause of intestinal infection related to antimicrobial drug consumption (*2*). Clinical manifestations range from mild to severe diarrhea, pseudomembranous colitis, toxic megacolon, sepsis, and ultimately death. Risk factors for CDI include duration of hospital stay, underlying illness, age (*3*), and previous use of virtually any antimicrobial drug, most frequently cephalosporins and fluoroquinolones (*4**–**10*).

The hypervirulent fluoroquinolone-resistant *C. difficile* PCR ribotype 027 North American pulsed-field type 1 (NAP1) (REA type BI, toxinotype III) has received attention as the cause of increasingly severe outbreaks and higher death rates, longer hospital stays, and frequent relapses (*8**,**9**,**11**,**12*). However, whether it really causes increased severity is questionable. Characteristics observed by previous studies may be due to selection bias or to the procedures used for diagnostic testing and reporting of cases; disease severity was similar in 2 groups of patients (PCR ribotype 027 and non-027) when recruitment to the study was done without reference to clinical signs and symptoms or PCR ribotype (*13*).

The pathogenicity of *C. difficile* is based on the action of at least 1 of the 2 main cytotoxins (A and B) acting as glycosyltransferases that modify guanose triphosphatases within the intestinal epithelial cells and lead to the disruption of the actin cytoskeleton. A recent study, which used a gene knock-out system, reinforced the fact that toxins A and B are comparable in terms of virulence, as shown by in vitro cytotoxicity and virulence in vivo (*14*). A binary toxin *C. difficile* transferase is found in some strains and belongs to the actin-modifying adenide dinucleotide protein–ribosyltransferases, which also impair the structure of actin cytoskeleton in epithelial cells (*15**,**16*). The pathologic significance of binary toxin is not yet clear. However, a recent study reports that binary toxin not only affects the actin cytoskeleton but also induces the formation of microtubule-based protrusions on the surface of epithelial cells, leading to increased adherence of bacteria (*17*).

Cultures positive for *C. difficile* are notifiable by the diagnostic laboratories in Denmark as part of the surveillance for gastrointestinal infections; in addition, isolates are selected under certain criteria and submitted to the National Reference Laboratory at Statens Serum Institut for further typing. The aim of the present study was to determine the case-fatality rate after diagnosis with *C. difficile*, according to toxin profile and PCR ribotype.

## Methods

### Surveillance System and Registries

**Figure 1:**
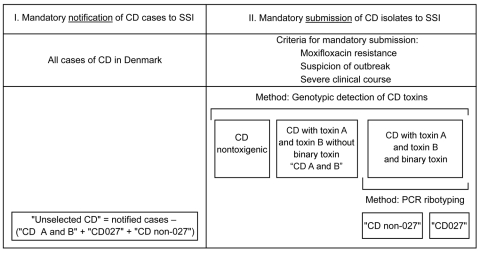
Description of *Clostridium difficile* (CD) infections surveillance in Denmark, with the 4 groups of *C. difficile*–infected patients included in the study, week 1, 2008–week 22, 2009. SSI, Statens Serum Institut; R, resistance.

All entries to 3 national registries in Denmark (the Danish Civil Registration System, the national Registry of Enteric Pathogens, and the *C. difficile* Microbiological Database) use a unique person registration number. These identifiers were used for the study. The study was retrospective, and we used a cohort design in which 4 groups of case-patients with *C. difficile* infection (Figure 1) were monitored from the date of diagnosis until the date of death or date of extraction from the registry. The study was conducted during week 1 of 2008 through week 22 of 2009. The unique patient identifier was used to link the registries. None of the registries contain clinical data.

The Danish Civil Registration System contains demographic information on all residents of Denmark and was used to retrieve the date of death. This registry does not contain information on the cause of death. The national Registry of Enteric Pathogens includes weekly case-based notifications of cultures positive for *C. difficile* from all departments of clinical microbiology of regional hospitals in the country. A second case-based database, the *C. difficile* Microbiological Database, which is separate from the Registry of Enteric Pathogens, contains information on isolates that undergo genotypic toxin detection and PCR ribotyping at the National Reference Laboratory at Statens Serum Institut, Copenhagen. Isolates are forwarded by departments of clinical microbiology if they are resistant to moxifloxacin, if severe clinical course is observed, or if an outbreak is suspected. These criteria were established in 2007, when sporadic cases of *C. difficile* PCR ribotype 027 were found for the first time in Denmark (*7*). They were reinforced in 2009, when the country experienced the first large *C. difficile* PCR ribotype 027 outbreak, which involved different hospitals of the Copenhagen Capital Region (*18*). Information on which specific criteria were used for submission of the individual isolates for subtyping was not available. No laboratory standard for primary diagnostics of CDI has been developed at the national level, and clinical microbiology departments use different methods, including environmental impact assessment, culture, PCR, or standard cytotoxin assays.

All isolates referred to Statens Serum Institut are genotyped to detect genes for the 3 toxins (A and B, and binary toxin). PCR ribotyping is subsequently performed on isolates possessing the genes for all 3 toxins (Figure 1). The methods used for genotyping of toxins and PCR ribotyping have been described in detail elsewhere (*19**,**20*). This study was approved by the Danish Data Protection Board.

### Definitions

Patients were assigned to 4 groups, depending on the characteristics of the isolates (Figure 1). Infected patients with an isolate possessing genes for toxins A and B and binary toxin were categorized either as *C. difficile* PCR ribotype 027 (CD027) or *C. difficile* PCR ribotype non-027 (CD non-027). A third group included patients infected with a strain possessing genes encoding for toxins A and toxin B, but not the binary toxin genes (CD A and B). A fourth group was created by subtracting the other 3 groups from patients with *C. difficile* infection that were notified to the surveillance laboratory system. Therefore, such patients were infected with isolates not referred for typing, presumably because the criteria for submission were not fulfilled. We refer to this group as unselected *C. difficile* unselected (CD-unselected).

Only the first episode of infection of the patient was considered. The first episode of CD027 overruled the first episode of CD non-027; the first episode of CD non-027 overruled the first episode of CD A and B; and the first episode of CD A and B overruled the first episode of unselected *C. difficile* infection. Therefore, the final dataset included only 1 observation per patient. The date on which the stool sample was collected was defined as the date of diagnosis.

### Statistical Methods

Kaplan Meier survival curves were created to determine the effect of time after diagnosis on the risk for death. Differences between curves were compared by using the log-rank test. Multivariate Poisson regression was used to estimate the risk ratio of death within 30 days after diagnosis. For survival analysis, patients were categorized into 2 groups, according to the presence or absence of binary toxin. Analysis was performed with STATA version 10 (StataCorp, College Station, TX, USA). Case-patients for whom 30 days of follow-up after infection could not be completed were excluded from the analysis (163 cases).

## Results

After the 2 microbiological datasets were merged, 2,299 case-patients with a first episode of infection were identified for the 17-month study. Of the 2,299 case-patients, isolates from 477 were referred to the national laboratory and were genotyped for toxins; of these 265 had genes for toxin A, toxin B, and binary toxin and were further ribotyped by PCR. Therefore, the 4 groups of patients with *C. difficile* infection used for the study consisted of 1,822 CD unselected, 212 CD A and B, 193 CD027, and 72 CD non-027. None of the isolates were positive for genes encoding only toxin A or B. The group of 72 CD non-027 consisted of 24 *C. difficile* PCR ribotype 078 (33%), 26 *C. difficile* PCR ribotype 66 (36%), and 22 *C. difficile* PCR ribotype 23, together with 9 other PCR ribotypes (31%).

Gender was equally distributed among the 4 groups of patients with CD unselected, CD 027, CD non-027, and CD A and B. The proportion of case-patients <50 years of age was much higher in the group with CD unselected (27.1%), compared with that of groups CD027 (4.6%) and CD non-027 (9.7%), which had more case-patients >80 years of age (Table 1). Most of the CD unselected*,* CD A and B, and CD non-027 were submitted by local clinical microbiology laboratories from areas not including the Capital region; most CD027 occurred in the Capital region, where the outbreaks of CD027 occurred in 2008–2009 (Table 1).

**Table 1:** Characteristics of case-patients according to group of *Clostridium difficile* infection, week 1, 2008–week 22, 2009, Denmark

Characteristic	No. (%) CD unselected, n = 1,822		No binary toxin		Presence of binary toxin	
			No. (%) CD A and B, n = 212		No. (%) CD 027, n = 193	No. (%) CD non-027, n = 72*
Male sex	796 (43.7)		100 (47.2)		87 (45.1)	31 (43.1)
Age group, y						
<50	494 (27.1)		30 (14.2)		9 (4.6)	7 (9.7)
50–59	166 (9.1)		14 (6.6)		8 (4.1)	8 (11.1)
60–69	280 (15.4)		33 (15.6)		25 (13.0)	14 (19.4)
70–79	367 (20.1)		70 (33.0)		52 (26.9)	16 (22.2)
>80	514 (28.2)		65 (30.7)		99 (51.3)	27 (37.5)
Region of local microbiology laboratory						
Capital region	263 (14.4)		46 (21.7)		164 (85.0)	22 (30.5)
Other parts of Denmark	1,502 (83.4)		158 (74.4)		29 (15.0)	50 (69.4)

*Consisting of *C. difficile* (CD) PCR ribotype 078 (n = 24), PCR ribotype 066 (n = 26), and PCR ribotype 023 and others (n = 22).

The case-fatality rate 30 days after diagnosis was independent of PCR ribotype in patients infected with strains that were positive for the binary toxin. More specifically, 54/193 case-patients with CD027 (28.0%, 95% confidence interval [CI] 21.8–34.9), and 20/72 case-patients with CD non-027 (27.8%, 95% CI 17.9–39.6) died within 30 days after infection. Case-fatality rate was 17.0% (36/212) for the group infected with CD A and B (95% CI 12.2–22.7) that did not possess genes for binary toxin, and lower (13.6%) for the 247/1,822 case-patients infected with CD unselected (95% CI 12.0–15.2). Among patients with CD non-027, seven deaths (29.2%) in CD078 were reported, 8 deaths (30.8%) in CD066, and 5 deaths in the group of other PCR ribotypes. No statistically significant difference was found between these case-fatality rates.

**Figure 2:**
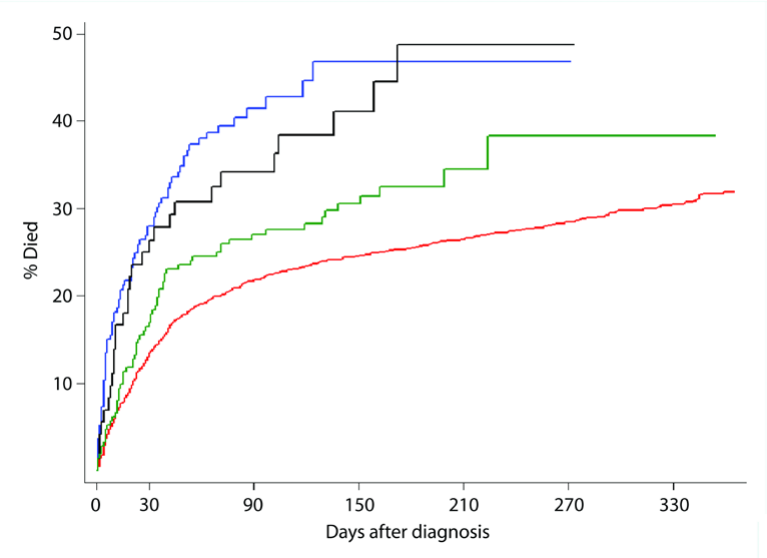
Kaplan Meier curves showing the probability of patient survival after diagnosis of *Clostridium difficile* infection according to the 4 different infection groups (log-rank test, p<0.001). Blue line, *C. difficile* PCR ribotype 027; black line, *C. difficile* PCR ribotype non-027; green line, *C. difficile* with toxins A and B without binary toxin; red line, *C. difficile* unselected strains not referred for typing.

Kaplan Meier curves were created for 1 year after diagnosis. A steep increase was seen in the case-fatality rates within 30 days after the diagnosis for all groups of patients, but especially evident for the 2 groups possessing the binary toxin genes (Figure 2). The shape of the curve for case-patients with binary toxin genes (CD027 and CD non-027) almost overlapped in the first 30 days; curves for the other 2 groups had a different shape (log-rank test, p<0.001). The curve of the group of patients infected with CD A and B showed an intermediate case-fatality rate as compared with the 2 groups with binary toxin and CD unselected. The cumulative risk of death (Kaplan Meier function) after 60 days was 18.4% in case-patients with CD unselected (336/1,822), 24.5% in those with CD A and B infection (52/212), 37.1 % with CD027 (71/193), and 30.5% with CD non-027 (22/72). After 90 days, the cumulative risk of death rose to 20.9% for CD unselected (381/1,822), 26.8% for CD A and B (57/212), 38.9% for CD027 (75/193), and 36.1% for CD non-027 (26/72). Kaplan Meier curves were also created after excluding all case-patients <50 years of age for all 4 groups (550 case-patients) because of the higher proportion of patients <50 years of age in the group of CD unselected strains. The curves showed a similar shape as compared when using the full dataset (figure not shown, log rank test, p<0.001). The cumulative case-fatality rate at 30 days also remained comparable: 18.1% for case-patients with CD unselected strains (239/1,319), 19.8% for CD A and B (36/182), 29.0% for CD027 (53/183), and 29.2% for CD non-027 (19/65).

On the basis of these observations, which showed a similar case-fatality pattern for the groups that possessed the genes for the binary toxin, in the regression analysis, we combined these 2 groups with the genes for the binary toxin (CD027 and CD non-027) into 1 group, and compared it with the group not possessing the binary toxin (CD A and B). Therefore, in the regression analysis, the group of CD unselected isolates was excluded because these isolates were not submitted for characterization.

Univariate analysis showed that the relative risk (RR) for death within 30 days after diagnosis was 1.8 (95% CI 1.2–2.7) for case-patients infected with *C. difficile* that possesses the genes for binary toxin in addition to toxin A and B, as compared with those infected with strains possessing only genes for toxin A and B, which provided the reference level (Table 2). Multivariate analysis, after adjustment for age, sex, and region, showed that the RR became 1.6 (95% CI 1.0–2.4) for case-patients infected with the strains encoding the genes for the binary toxin when compared with the reference group of patients infected with strains without the genes for binary toxin (Table 2).

**Table 2:** Relative risk for death within 30 days after diagnosis of *Clostridium difficile* infection, univariate and multivariate analysis, week 1, 2008–week 22, 2009, Denmark*

Variable	No. deaths	Crude risk ratio (95% CI)	Adjusted risk ratio (95% CI)
CD A and B	36	Reference	Reference
CD 027 + CD non-027	74	1.8 (1.2–2.7)	1.6 (1.01–2.4)
Male sex	47	0.9 (0.6–1.2)	1.0 (0.7–1.4)
Age group, y			
<50	1	Reference	Reference
50–59	3	4.9 (0.5–47.2)	4.5 (0.5–43.9)
60–69	9	6.2 (0.8–48.8)	6.0 (0.8–47.3)
70–79	37	14.5 (2.0–105.8)	13.8 (1.9–100.9)
>80	60	17.4 (2.4–125.3)	15.5 (2.1–112.6)
Region			
Capital	63	1.4 (1.0–2.1)	0.9 (0.6–1.2)
Other parts of Denmark	47	Reference	Reference

*CI, confidence interval; CD, *Clostridium difficile*.

## Discussion

We used surveillance data to describe the case-fatality rate after a diagnosis of *C. difficile* infection. We found that the case-fatality rate is highest after infection with strains that possess genes for the binary toxin in addition to toxins A and B, irrespective of the PCR ribotype. Strains encoding genes for toxins A and B, but not binary toxin, showed a lower case-fatality risk.

A number of studies have addressed the issue of risk for death and severity of disease after infection with *C. difficile*. Overall, *C. difficile* PCR ribotype 027 has been associated with more severe disease and increased death rates. Nevertheless, many studies did not have a strict sampling frame or appropriate epidemiologic design, and their findings have been questioned by recent evidence (*13**,**21*). Our results are consistent with the initial findings that *C. difficile* PCR ribotype 027 is associated with elevated risk of death, but we elaborate further on the molecular characterization according to toxin profile. We suggest that the previously observed high case-fatality rate observed in *C. difficile* infection cannot be solely ascribed to excess risk for death after infection with PCR ribotype 027; other markers of virulence may be more appropriate than the PCR ribotype itself. The inclusion of case-patients on the basis of clinical findings only (*1**,**8**,**9**,**12*), the different criteria used to select strains for PCR ribotyping (*13**,**21**–**23*), or the lack of differentiation in separate groups according to toxin profiles (*24*) might have accounted for variation of estimates across the studies, as well as an overestimation of the risk for death associated with *C. difficile* PCR ribotype 027.

We observed a 28% case-fatality rate at 30 days for the 2 groups possessing the binary toxin: estimates from previous studies in Canada indicated a risk for death of 23% for patients with *C. difficile*–associated disease (CDAD), in a hospital in which *C. difficile* PCR ribotype 027 strain made up two-thirds of the isolates (*12*); or of 25% in another study involving 12 hospitals in which case-patients with CDAD were compared with controls without CDAD. In the latter study, 129/157 strains examined had pulsed-field gel electrophoresis patterns identical to NAP1 (*8*). In the Netherlands, 12.9% lethality was reported for *C. difficile* PCR ribotype 027 as compared with 7.0% in other *C. difficile* PCR ribotypes non-027 (*21*).

A few clinical studies indicate that the production of binary toxin correlates with the severity of CDI, rendering the strains with binary toxin more virulent. A case–control study conducted in 2005 included 26 patients infected with strains producing binary toxin in addition to toxins A and B and 42 controls infected with strains producing toxins A and B only. Diarrhea in case-patients was more frequently associated with abdominal pain (61.5% vs. 26.2%; p = 0.003) and with liquid stools (76.9% vs. 59.5%; p = 0.14) (*25*). Another case–case study from 2007 confirmed this tendency, showing that binary toxin–positive strains were significantly associated with more severe CDI (RR 3.38, 95% CI 1.29–8.85) and with higher case-fatality rates (RR 2.55, 95% CI 1.25–5.21) (*26*). Binary toxin–positive strains that produced neither toxins A and B were investigated in the rabbit ileal loop model to elucidate the contribution of binary toxin in the pathogenesis of CDI (*27*). This study showed that binary toxin contributed to marked nonhemorrhagic fluid responses when responses of nontoxigenic strains were compared. However, strains that produced toxins A and B gave rise to hemorrhagic fluid responses in this assay. In the same study, challenge with clindamycin-treated hamsters resulted in colonization of the binary toxin–positive strains but not diarrhea and death as seen for the strains that produced toxins A and B. Therefore, binary toxin may play an adjunctive role in the pathogenesis of disease caused by strains positive for toxins A and B (*27*).

Historically, *C. difficile* infection was not considered a severe disease, and studies performed 15 years ago reported case fatality rates of 3.0%–3.5% (*28*,*29*). Due to the current laboratory surveillance system, we were able to quantify 30-day case-fatality rate of a reference group (CD unselected isolates not referred for typing) at 14%, which provides an updated estimate of such baseline category. In a registry-based study in Finland, performed before *C. difficile* PCR ribotype 027 was identified in the country for the first time, a 14.2% 30-day death rate was reported among those discharged with a CDAD-related diagnosis (*30*). In Quebec, 13.8% of deaths reported 30 days after CDAD diagnosis were observed at the beginning of the *C. difficile* PCR ribotype 027 epidemic in 2003 (*9*).

Many studies have reported that a consistent fraction of the deaths occurring after *C. difficile* infection will be attributable to the bacterium (*1**,**8**,**12**,**21**–**23**,**31*) and that attributable death increases linearly with age (*8**,**31*). In our study, we could not differentiate between death after infection and attributable death because the registries did not contain information on the cause of death nor underlying illness. An excess proportion of deaths caused by CD027 and other strains with binary toxin corroborates recent evidence from Canada, which showed an increased risk for death in patients infected with the NAP1 strain (*24*)

Due to the availability of the national registries, we were able to investigate the case- fatality rate for a large cohort of patients and to get statistically significant results when investigating groups with different toxin profiles of the same infection. In addition, we performed multivariate analysis adjusting for age, sex, and region. Multivariate analysis indicated that the risk of death was increased by 60% (RR 1.6) for the strains possessing the binary toxin, irrespective of age, sex, and region of the laboratory submitting the isolates. Use of the registries made it possible to design the study on an individual patient basis, not only on isolates, and made it unlikely that deaths were missed.

The main limitations of the study were that we were not able to collect data on underlying illness from the registries and that the toxin gene profile of the unselected isolates not referred for further typing was not characterized. We accounted for the latter possible bias by excluding this group in the regression analysis, and by using the group toxin profiled without genes for binary toxin (CD A and B) as the reference level. The lack of availability of data on underlying illness means that the long-term case fatality explored with the Kaplan Meier survival function must be interpreted with caution. However, our estimates at 3 months after infection were comparable to those of a previous study in which confounding caused by underlying illness was addressed (*12*). Therefore, *C. difficile* could play a role in risk for death in the longer term. An increase in long-term deaths after bacterial gastrointestinal infections has been observed (*32**,**33*). Complications of operations performed after toxic megacolon, disruption of the colonic flora and intestinal cells, subsequent malabsorbtion, and, most importantly, the recurrence of infection, could be some of the mechanisms involved in long-term deaths after infection with *C. difficile*. About 19%–20% of first episodes of infection with *C. difficile* will be followed by a recurrence (*34*), either due to a relapse or reinfection with another strain.

In conclusion, our registry-based study demonstrates that patients infected with *C. difficile* strains possessing the binary toxin genes and genes encoding toxins A and B have a higher 30-day case-fatality rate, irrespective of PCR ribotype, when compared with strains that have toxins A and B only. Early recognition of the toxin profile might be beneficial in terms of clinical management of the disease. Future studies should address whether the binary toxin or an unknown co-expressed factor might be responsible for increased case-fatality rates. *C. difficile* PCR ribotype 027 can no longer be considered the only PCR ribotype associated with severe disease, and efforts to control CDI should target all virulent strains of *C. difficile*, not only *C. difficile* PCR ribotype 027**.**
